# Hematological and biochemical profiles, infection and habitat quality in an urban rat population

**DOI:** 10.21203/rs.3.rs-4817847/v1

**Published:** 2024-09-11

**Authors:** Ticiana Carvalho-Pereira, Gabriel G. Pedra, Daiana S. de Oliveira, Fabio N Souza, Caio G. Zeppelini, Luana R. N. Santos, Ricardo D. Couto, Thiago C. Bahiense, Eduardo M. da Silva, Michael Begon, Mitermayer Galvão Reis, Albert I. Ko, James E. Childs, Federico Costa

**Affiliations:** Federal University of Bahia; University of Liverpool; Instituto Gonçalo Moniz, Fundação Oswaldo Cruz, Ministério da Saúde; Federal University of Bahia; Federal University of Bahia; Instituto Gonçalo Moniz, Fundação Oswaldo Cruz, Ministério da Saúde; Federal University of Bahia; Federal University of Bahia; Federal University of Bahia; University of Liverpool; Instituto Gonçalo Moniz, Fundação Oswaldo Cruz, Ministério da Saúde; Yale University; Yale University; Federal University of Bahia

**Keywords:** host condition, hematological profile, hormone-biochemical related stress, Rattus norvegicus, helminth species, Leptospira interrogans, infection, habitat quality

## Abstract

Host condition is key in understanding disease dynamics. In an urban population of Rattus norvegicus, we aimed to assess whether infection of Leptospira interrogans and helminths was associated with patterns of host hematological and hormone-biochemical stress-related conditions. Rat kidney imprints and urine were used to identify and quantify L. interrogans, and feces samples for helminth eggs and corticosterone metabolites. Blood samples were taken for complete blood counts and specific biochemicals in rats’ sera. Principal Component Analyses were performed to check whether rats would be grouped according to health profiles. We obtained hematological and hormone-biochemical data from 95 and 61 rats, respectively. Hematological PCA revealed distinct rat groups: typical (T), eosinophil deficient (Eos-D), eosinophil- and monocyte- deficient (EM-D) and monocyte deficient with high immature neutrophils (Mon-D). No association between L. interrogans or helminths and rat health profiles was observed, except with Trichiuridae, which mean intensity was significantly higher when all deficient groups were pooled together compared to the T-group. The poorest condition group was found in areas with fewer rat burrows than the T-group, indicating EM-D had a reduced ability to occupy “good” quality habitats. In natural populations, hematological profiles may reflect host’s overall condition, instead of responses to specific infections.

## Introduction

Parasitic co-infection is commonplace and has garnered increasing interest, due to the push for integrated disease control [1–4]. Multiple concurrent infections form complex synergistic/antagonistic relationships through competitive interaction for similar resources or biotopes [5, 6]. Griffiths, Pedersen [7], constructing a summary network for human co-infection, suggested that indirect interactions – mainly related to resource consumption (exploitation competition), and host immune responses (“apparent competition”) [8] – are more likely to structure parasite interactions than expected by chance. Host condition may play an important part in modulating co-infection by influencing available resource quality for parasites, as well as the host’s ability to mount an immune response. Also, previous poor condition may increase individual susceptibility to infection, further weakening their condition, possibly leading to co-infection in a vicious circle [9]. Iindividuals in poor condition, after infected, are more likely to carry greater infection burdens, further weakening their defenses and condition [10]. At the population level, this may manifest as greater pathogen shedding into the environment [11]. Therefore, host susceptibility becomes key to understand infection dynamics in natural populations.

Host hematological profile can be indicative of condition and immunologic investment, which may also suggest responses to previous/current infections [12]. Low red blood cell (RBC) counts characterize anemia, which can occur due to poor nourishment or infection [13–15]. High lymphocyte counts in the bloodstream indicate acquired immunity, while neutrophils and monocytes indicate acute and chronic inflammatory responses, respectively [16]. High eosinophil counts are expected in macro-parasite infections [16, 17]. Conversely, low leukocyte leveçs can be associated with increased levels of glucocorticoids, after chronic stress [18].

Natural host populations constantly face stress in the form of environmental unpredictability, physical and social challenges, which can reflect in reduced conditions and vulnerability to infection [19]. Stress hormones, such as glucocorticoids, regulate glucose metabolism to prepare the organism for imminent activity [20]. These hormones are closely involved with the immunity, controlling inflammatory responses [21]. As metabolites excreted in feces, glucocorticoids are good indicators of chronic stress, as feces accumulate circulating levels for hours [22–24].

The Norway rat can host different micro/macro-parasite species usually found in co-infection [6, 25–27], and is the main reservoir of *Leptospira interrogans*, one of the etiological agents of leptospirosis [28]. Co-infection has been shown to influence parasite infection risk and intensity in urban rat populations [6]. Herein, we assessed 1) whether infection and infection intensity of *L. interrogans*, and digestive tract helminths, were associated with host condition variations, assessed by hematological and stress hormone (corticosterone metabolites) profiles, in an urban *Rattus norvegicus* Berkenhout (1769) population; 2) whether there were discernable variation patterns in field, by checking associations between host profiles and environmental and demographic variables.

## Materials And Methods

### Sampling Design

The study area was in Pau da Lima (13°32’53.47” S; 38°43’51.10” W), a peripheric community in the city of Salvador (BA – Brazil). The area (0.17 km²) is characterized by irregular urban occupation within three geographic valleys, high-density human occupation, and poor sanitation conditions (e.g., open sewers and lack of refuse collection) [29, 30].

Norway rats were captured using Tomahawk-like traps (45 × 16 × 16 cm) placed at 101 sampling points, generally located in residents’ backyards, following previous protocols [31, 32]. For each rat, we sectioned the inferior vena cava and collected a blood sample in EDTA tubes (2 mL). Subsequently, we collected another blood sample and two fecal samples, one directly from the intestines, and later stored at −20°C, for corticosterone metabolites analysis. Fecal samples were collected to identify and quantify eggs of helminth species, placed in 10% formalin. We collected kidney imprints and urine to identify and quantify *L. interrogans*.

### Ethics declaration

Animal and environmental sampling (including rat demography and body condition) followed previously validated protocols [27, 33–36]. This study was approved by the Ethical Committee of Animal Use (CEUA) protocol #003/2012 of Oswaldo Cruz Foundation (Fiocruz). All methods were carried out in accordance with relevant guidelines and regulations. All methods are reported in accordance with ARRIVE guidelines.

### Blood Data Collection

Red blood cell (RBC) counts were automatically performed by the ABX Micros ESV 60. Total and differential White Blood Cell (WBC) counts were performed by differentiating 100 cells, in the cell monolayer area, from air-dried and Diff-quick stained blood smears by the Panotic method (Laborclin^®^, Pinhais, PR, Brazil). We evaluated globulins, albumin and free fatty acids concentrations in rat serum, to account for their covariate effect when analyzing the stress hormone profile. Total cholesterol, Triglycerides, Total proteins and Albumin in sera concentrations were automatically estimated by the Selectra E. auto analyzer, with specific reagents provided by Labtest, following manufacturer’s instructions (assay kit reference numbers: 76, 87, 99, 1007, respectively). Globulin concentrations were obtained by calculation (total proteins - albumin concentrations).

Fecal corticosterone metabolites concentrations were estimated by first weighing 0.05 g of each sample, previously defrosted and homogenized. Then, we quantified corticosterone metabolites using the Cayman Chemicals Corticosterone ELISA kit (Cayman Chemical No. 500655). The concentrations obtained were converted to ng/g of feces following Hoby, 2015.

We applied Hoffman, Pons [37] sedimentation, and Gordon and Whitlock [38] flotation techniques to the 10% formalin fecal samples to, respectively, identify and quantify (EPG) helminth species eggs. Immunofluorescence and real-time PCR (qPCR) were performed with the kidney imprints and urine samples to identify and quantify (GEq) *L. interrogans* [27, 36].

### Statistics

We estimated median, interquartile, and full ranges of each hematological, serum biochemicals, and hormonal variable collected. Principal Component Analyses (PCA) were performed with two separate sets of health condition variables, initially, to check whether rats would be separated into groups according to different hematological or hormonal-biochemical patterns, referred to hereafter as health profiles. The first set considered only blood cells, representing physio-immunological conditions. The second set included corticosterone metabolites and specific serum biochemicals measured, being related to stress. These PCAs will be referred to hereafter as Bc-PCA and Cort-PCA, respectively. All variables were log-transformed prior to the analyses, with PCAs performed with a covariance matrix. For each PCA, the Keiser-Guttman criterion was applied to identify the most meaningful axes. Then, a Ward’s Minimum Variance Clustering was carried out to identify groups of rats closely related according to Borcard, Gillet [39].

To check whether groups of rats were associated with infection with each helminth species or *L. interrogans* individually, we performed chi-squared or Fisher’s exact tests (expected frequencies <5) with contingency tables [40]. Where initial results were, at least, marginally statistically significant (*p*<0.1), we applied a series of eliminations of rows and columns of the contingency tables, whilst re-running the significance test, to identify the levels of the two variables with which the pattern was associated. We also investigated associations with relevant combinations of groups, where appropriate. Additionally, we investigated whether mean intensity of infection (only positive individuals) of each helminth species or *L. interrogans* individually varied between groups of rats by permutation ANOVA, using Fisher’s least significant difference (LSD) method post-hoc test was used as applicable. Because glucocorticoids can influence the concentration of white blood cells, we checked whether fecal corticosterone metabolites were associated with Bc-PCA groups as well. To account for environmental and host demographic variations in the field, we used the same statistical tests to check whether rat groups (or combinations of groups) were associated with geographic valleys, proxies of rat density, and biotic variables (sex, age, maturity, scaled mass index (Smi), presence of wounds and internal fat, see Table S1). In cases where two or more variables were independently associated with the PCA groups, combined effects were assessed with MANOVA. All analyses were performed in R [41], using the packages ‘*vegan*’ and ‘*lmPerm*’, considering significance level of *p* < 0.05.

## Results

**Figure 1:**
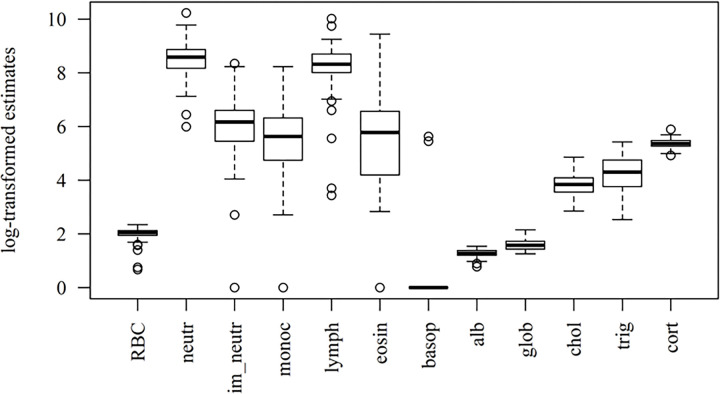
Boxplots of the hematological and hormone-biochemical stress profiles in urban Norway rats. ‘neutr’ = neutrophils; ‘im_neutr’ = immature neutrophils; ‘monoc’ = monocytes; ‘lymph’ = lymphocytes; ‘eosin’ = eosinophils; ‘basop’ = basophils; ‘alb’ = albumin; ‘glob’ = globulins; ‘chol’ = cholesterol; ‘trig’ = triglycerides; ‘cort’ = corticosterone metabolites.

A total of 120 rats were captured, with complete hematological information obtained for 95 individuals, and complete hormone and serum biochemistry assessment performed for 61. The summary of health condition variables is presented in Table 1 and Fig. 1. Among the leukocytes, neutrophils and lymphocytes presented the highest numbers, whilst presenting the least variation within the population. Likewise, RBC counts, and fecal corticosterone metabolite estimates varied little among rats. Only two individuals presented non-zero basophil counts and, for lack of representativeness, this cell type was disregarded for the PCA.

Of the two PCAs performed, Bc-PCA presented two significant axes (explaining together 82% of variation), whereas Cort-PCA presented only one (Fig. S1A-B). Because the first axis of Cort-PCA only accounted for 70% of the variation, we also included PC2 to aid interpretation, as together they explained 89% of the variation.

**Figure 2:**
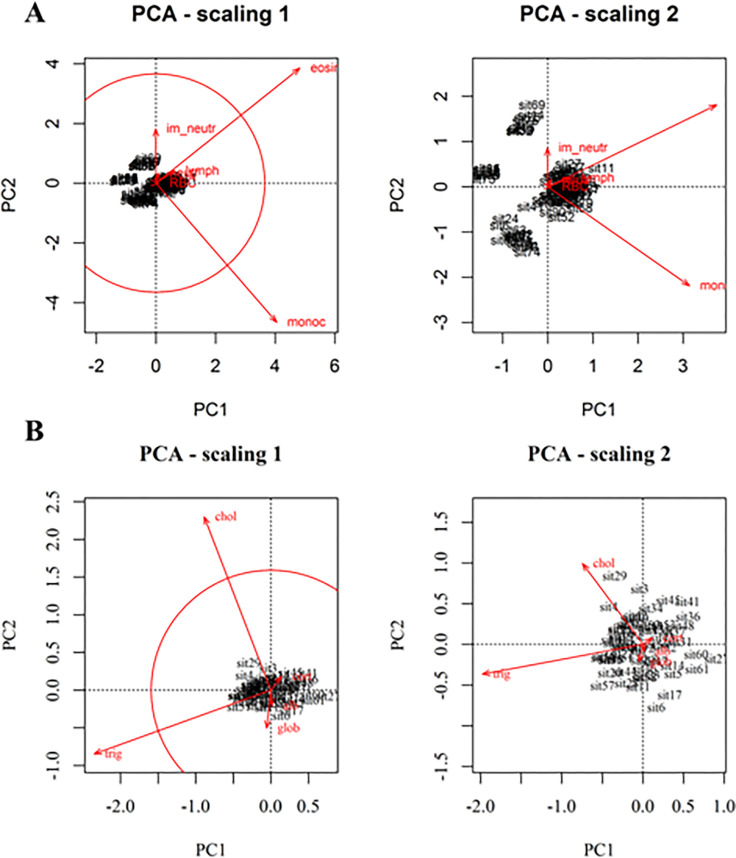
Bc-PCA biplots (**A**) and Cort-PCA biplots (B), drawn with function *cleanplot.pca*(). The *circle of equilibrium contribution*, in the ‘scaling 1’ biplot, highlights the higher contribution of eosinophils and monocytes in **A**, and contribution of triglycerides and cholesterol in **B**, in explaining the variation of the data.

For the Bc-PCA, eosinophils and monocytes were the two variables with higher-than-average contributions in explaining the variation (Fig. 2 – scaling 1). Eosinophils and lymphocytes were positively correlated with one another, while monocytes and immature neutrophils were negatively correlated (Fig. 2 – Scaling 2). The Bc-PCA presented four clearly distinct rat groups (Fig. S2A), characterized by different hematological profiles. The group with the highest number of individuals is denoted typical (T) and included individuals within the normal ranges of blood cells found for reference rats, together with individuals capable of mounting immune responses (e.g., neutrophilia and eosinophilia). The other three groups are: eosinophil deficient (Eos-D), eosinophil and monocyte deficient (EM-D), and monocyte deficient with high immature neutrophil numbers (Mon-D).

**Figure 3:**
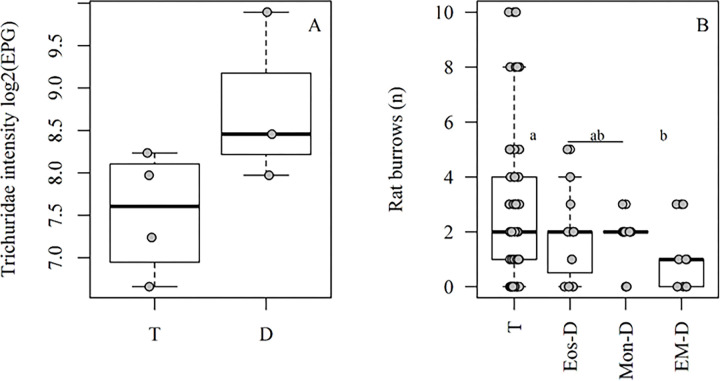
Variables associated with Bc-PCA groups. A) Trichiuridae intensity (log2(EPG)) vs Bc-PCA groups. B) Number of rat burrows vs Bc-PCA groups. ‘D’ = for the pool blood cells deficient groups (Eos-D, Mon-D and EM-D). Different letters mean statistical significance by Fisher LSD post-test.

The Cort-PCA did not present any apparent grouping (Fig. 3). Triglycerides and cholesterol accounted for the highest contribution in explaining variation in the data (Fig. 3 – Scaling 1). Triglycerides were negatively correlated with fecal corticosterone metabolites, which, in turn, showed less importance for the ordination of the rats in the plane (Fig. 3 – Scaling2). Here, we restricted cluster grouping to the minimum possible, two, defined by the dendrogram (Fig. S2B).

We identified five species/groups of helminths with prevalence ≥10% to seek associations between the PCA groups: *Strongyloides* sp. (95%), *Nippostrongylus brasiliensis* (57%), *Angiostrongylus cantonensis* (33%), *Hymenolepis* spp. (14%) and Trichiuridae (12%). We found 72% prevalence of *L. interrogans*, also included in the analyses.

The distribution of rats among the Bc-PCA groups was independent of infection by any species identified in this study, except for a significant increase in Trichiuridae intensity, when all deficient groups were compared to the typical (*p* < 0.05, Fig. 3A). Neither demography nor body condition variables were associated with the groups. Mean concentration of fecal corticosterone metabolites did not vary between groups. Occurrence of rats in the Bc-PCA groups was marginally associated with location, (i.e., the three valleys, *p*= 0.08). Fewer rats from the T group were found in Valley 1, whereas rats from the EM-D group were more frequently found there. The mean number of rat burrows (proxy for rat density/environment quality) was significantly lower where EM-D individuals were caught, compared to the typical group (*p* = 0.02; Fig. 3B). These associations were independent (mean number of rat burrows did not vary with valleys, *p* = 0.52). No combined association between mean number of rat burrows and valleys was found (Pillai’s Trace: 0.11, F_(3,91)_ = 1.84, *p* = 0.09; Rat burrows: F = 2.06, *p* = 0.11; Valleys: F = 1.84, *p* = 0.15). None of the variables were associated with the Cort-PCA groups.

## Discussion

The urban rat population here assessed contained groups of individuals in varying conditions of health, according to hematological profiles. In terms of indicators of hormone-biochemical-related stress, however, no patterns were found. Rat grouping, characterized by hematological profiles, might reflect differences habitat quality across the environment. Although helminth species and *L. interrogans* were generally in high prevalence in the study population, only the intensity of one helminth species was associated with rat groups.

Overall mean RBC count was low compared to reference values from laboratory rats [13, 42], with about 30% of the animals presenting indications of what has conventionally been considered anemia (mean count < 6.08 10^6^/μL) [17]. Beldomenico, Telfer [12] found similar results comparing wild and captive Microtus agrestis, with even high values among field population samples being lower than in the near-optimal conditions of an animal house (abundant food, negligible infection), suggesting that natural populations are generally resource- and/or energy-limited, and can only increase investment in, say, white cells by limiting investment in, for example, RBCs.

Total leukocyte average value, on the other hand, was higher than the average found for reference rats, with approximately 33% of the animals presenting even higher values than the maximum value found from 88 reference rats [42]. These values received the highest contribution from lymphocytes and neutrophils, indicating that urban rats consistently allocate resources to immune responses, particularly acute inflammation.

Neutrophils respond after tissue injury or bacterial infection with rapid increase in blood circulation [16]. Other white blood cells (monocytes, eosinophils, basophils) although represented in lower numbers, presented the highest variation among rats, with cases of eosinophils and monocytes reaching zero counts. Eosinopenia and monocytopenia can occur due to injury, malnutrition or be stress-hormone induced (e.g., epinephrine and ACTH) [14, 43, 44] all characterizing situations expected in nature.

Fecal corticosterone metabolites estimates were generally high within the population. Although there is variation in literature regarding baseline levels for unstressed reference laboratory rats (e.g., Amara, Cole [45], Pihl and Hau [46]), our mean corticosterone metabolites level is consistent with reported levels of stress or high activity [24, 47]. This suggests that urban rats are, in general, under stress, related to environmental, physiological, behavioral, infection [19, 48], or even anthropogenic causes [49]. Considering that this same rat population in a previous study exhibited 60.1% (231/384) of males with skin wounds and 47.8% (181/379) in females (Panti-May *et al*. 2016), a potential association between stress and individual condition.

Alternatively, high average concentrations might indicate acute stress due to caging, as the time delay between blood and fecal concentrations is ~12 hours [47], possibly reflecting the interval between capture and sample collection. Stress situations can also lead to increased triglyceride concentrations, which increase glucose availability against stress [21]. The fact that no clear groups were found within Cort-PCA multivariate space may indicate that animals were caught with differentiable hormone-biochemical profiles, but by the time of testing, the profiles (also) reflected an acute stress response.

On the Bc-PCA, rat groups characterized by either eosinopenia (Eos-D), monocytopenia with increased circulation of immature neutrophils (Mos-D) or both eosinopenia and monocytopenia (EM-D), may be in poorer condition, compared to the typical group (T). It is noteworthy that Mos-D, although monocyte deficient, presented high immature neutrophil numbers, indicating a regenerative process of increasing the number of neutrophils available to combat acute inflammations [16], a good host response. Individually, none of the Bc-PCA groups were associated with any of the infections assessed. However, when all poor condition groups were pooled, Trichiuridae intensity was significantly higher compared to T. Eucoleus sp., a Trichiuridae, is known to cause mucosal hyperplasia and submucosal inflammation in the non-glandular stomach of Norway rats [50]. Our finding might indicate that eosinophil/monocyte-deficient rats are more prone to higher Trichiuridae burdens by being incapable of mounting an immune response against them, as both eosinophil and monocytes (macrophages) may play a role [16, 51, 52]. Alternatively, high Trichiuridae intensities may have caused reduced circulation of these blood cells, particularly eosinophils, since *Trichuris muris* infection, for example, can modulate host immune response to the Th1 arm [53].

Compared to the T group, rats from the poorest condition group, EM-D, were caught in areas with lower rat densities, as indicated by an association with reduced numbers of burrows. This might indicate that rats from the EM-D group occupied lower quality habitat because, being in poor condition, they were excluded from high-quality habitat, or that their condition was itself a consequence of low-quality habitat. Among Norway rat social groups, stable colonies are structured primarily by dominance hierarchies and a network of differential relationships among members, allowing for reproduction success and decreased mortality due to disease/predation [54]. This, and the aggressive behavior of Norway rats [54], might have restricted the weaker competitors (e.g.: EM-D) to low-resource areas. The fact that mean number of burrows did not vary between valleys indicates heterogeneity in habitat quality across the study area. The difference in group occupation of valleys, however, indicates that valley 1 might provide fewer good-quality habitat sites than the other two valleys. Urban rat abundance varies within short geographical distances [55], with increases mostly associated with human conditioners [35]. Valley 1 has the lowest human density.

## Conclusions

Our study identified Norway rat groups in different health conditions among an urban population, co-infected by helminths and *L. interrogans*. Although no strong association between infection and health profiles was found, there was an indication that eosinopenic and monocytopenic rats can carry higher Trichiuridae burdens. Other infections reported for in *R. norvegicus* (e.g., Seoul virus, *Bartonella* sp., cysticercosis, hepatic capillariasis) [25, 27, 56] might be associated with these health profiles and should be further investigated. Alternatively, different genetic populations among *R. norvegicus* [57, 58] might be associated with the varying manifestations of white blood cell profiles observed, although further tests are necessary.

Host condition patterns reflected host population dynamics, specifically the occupation of high-quality habitats, which are apparently heterogeneous within the study area. Our study suggests that, in natural populations – with low resources, numerous infections and high stress – hematological profiles may be more a reflection of host overall condition, overriding direct responses to specific infection as seen in the (unnatural) laboratory.

## Figures and Tables

**Table 1 –:** Summary of health condition variables

Variables (Unity)	Mean (Std)	Median (IQR)	Range
Hemogram			
RBC (10^6^ /μL)	6.58 (± 1.32)	6.78 (6.02 – 7.36)	(0.96 – 9.46)
Leukocytes (10^9^ /L)	12.68 (± 7.63)	11.80 (8.60 – 14.25)	(0.70 – 62.70)
Neutrophils (10^9^ /L)	5.91 (± 3.74)	5.32 (3.52 – 7.08)	(0.40 – 27.59)
Immature Neutrophils (10^9^ /L)	0.64 (± 0.70)	0.47 (0.23 – 0.73)	(0.00 – 4.19)
Monocytes (10^9^ /L)	0.50 (± 0.70)	0.28 (0.11 – 0.55)	(0.00 – 3.75)
Lymphocytes (10^9^ /L)	4.91 (± 3.20)	4.08 (3.00 – 5.99)	(0.03 – 22.33)
Eosinophils (10^9^ /L)	0.59 (± 1.34)	0.32 (0.06 – 0.71)	(0.00 – 12.54)
Basophils (10^9^ /L)	0.005 (± 0.036)	0.00 (0.00 – 0.00)	(0.00 – 276.00)
Serum biochemistry			
Total cholesterol (mg/dL)	46.25 (± 19.55)	46.40 (33.20 – 55.70)	(16.30 – 127.30)
Triglycerides (mg/dL)	85.69 (± 49.61)	75.50 (46.00 – 116.10)	(11.60 – 224.80)
Total Proteins (g/dL)	6.65 (± 1.15)	6.60 (5.89 – 7.37)	(3.70 – 9.53)
Albumin (g/dL)	2.61 (± 0.45)	2.59 (2.35 – 3.01)	(1.18 – 3.36)
Globulins (g/dL)	4.04 (± 0.97)	3.86 (3.41 – 4.64)	(2.52 – 6.50)
Fecal corticosterone Metabolites (ng/g)	218.0 (± 37.6)	212.4 (192.6 – 238.8)	(136.4 – 362.1)

Std = Standard deviation; IQR = Interquartile range
